# The social role of pediatrics in the past and present times

**DOI:** 10.1186/s13052-021-01190-6

**Published:** 2021-12-18

**Authors:** Gregorio Serra, Mario Giuffrè, Ettore Piro, Giovanni Corsello

**Affiliations:** Department of Health Promotion, Mother and Child Care, Internal Medicine and Medical Specialties “G. D’Alessandro”, University of Palermo, Palermo, Italy

**Keywords:** Children, Children’s care, Pediatric hospitals, History of pediatrics, Pediatrician

## Abstract

Pediatrics and society are closely related. This link is as old as the history of Pediatrics, and dates to the second half of the eighteenth century. The vocation of the first European pediatric schools, indeed, was clinical and scientific, as well as social. The founding fathers of Pediatrics were scientists of great talent, and many of them benefactors and philanthropists. They spent their lives assisting the suffering childhood, and became promoters and organizers of social securities for the poorest and most vulnerable categories. The attention to the problems of abandonment was closely linked to study, prevention, and treatment of pathologies (especially infectious, deficiency and neurological ones). The profile and activity of pediatricians grew in the following decades after the birth of the first pediatric schools. The University institutions contributed to provide a further impulse to childcare as well as cultural authority, also thanks to the foundation of the first chairs and scientific journals of Pediatrics. The relevance and prestige of the studies performed rapidly spread throughout Europe, and also reached our country, contributing to a progressive and relevant improvement in the quality of children’s care, and in the meantime to the decrease of neonatal and infant mortality rates.

Today’s pediatricians, as in the past, must spend his efforts to face the needs of children and their families, be their social receptor, interpreter if necessary, and credible and authoritative interlocutor beside institutions. The current coronavirus pandemic dramatically exposed social inequalities and inequities. In this new scenario, the pediatrician’s role of defender of all children becomes even more necessary and indispensable. Here we trace the historical steps which led to the birth and development of pediatrics, as independent medical discipline with ethical and social vocation. Its rise within the University institutions is analyzed, as well as the contribution of the greatest European and Italian masters. Finally, the role of today’s pediatrician is described, his responsibilities also in dealing with new health critical issues, related to the biological, cultural, and psychological changes of the patients of present days. He must have holistic competences, to effectively take care of all children. In addition, he must socially act to guarantee the best possible context for the well-being of the child.

## Background

Pediatrics and society are closely related. This link is as old as the history of Pediatrics, and dates to the second half of the eighteenth century. The vocation of the first European pediatric schools, indeed, was clinical and scientific, as well as social. The founding fathers of Pediatrics were scientists of great talent, and many of them benefactors and philanthropists. They spent their lives assisting the suffering childhood, and became promoters and organizers of social securities for the poorest and most vulnerable categories [1]. The attention to the problems of abandonment was closely linked to study, prevention, and treatment of pathologies (especially infectious, deficiency and neurological ones) [2]. The profile and activity of pediatricians grew in the following decades. The University institutions, then, contributed to provide a further impulse to childcare as well as cultural authority, also thanks to the foundation of the first chairs and scientific journals of Pediatrics. The relevance and prestige of the studies performed rapidly spread throughout Europe [1, 2]. All these achievements, in addition to the increasing economic well-being and the improvement of the social and hygienic conditions, led to a progressive and relevant growth in the quality of children’s care, and in the meantime to the decrease of neonatal and infant mortality rates.

Here we trace the historical steps which led to birth and development of pediatrics as independent medical discipline, with intrinsic and unavoidable ethical and social vocation. The growth of this specialty is outlined, also analyzing its rise within the University institutions, with the foundation of the first chairs, and the contribution of the greatest European and Italian masters. In the second section of the text, the role which the pediatrician should currently play is described, especially his responsibilities beside institutions as well as in facing new growing health critical issues, related to the biological, cultural, and psychological changes of the patients of present days. Finally, the challenges which pediatricians are called to deal with are analyzed, oriented to guarantee to all children the best possible assistance, to fight against inequalities and poverty, as well as to protect their rights within families and their identity in the society in all its forms, personal, cultural, and religious.

### Pediatrics and society in the past

A radical turning point about the attention given to children, within pedagogy, literature, and family, occurred between the 18th and nineteenth century. A new educational concern appeared thanks to the thought and work of Jean Jacques Rousseau, the father of modern pedagogy. Since then, the value of childhood shifted from an economic plan to that of feeling, regarded as affection and attention. The idea of the new Man introduced and promoted by the Enlightenment, opened to the consideration of infancy as the starting dimension of men. Therefore, doctors and pedagogists began to share a body’s philosophy, which allowed children to be protected. The techniques on the body started to join character education. Indeed, authoritative thinkers of the eighteenth century observed and controlled the “infantile bodies” of their children. They “looked with special attention at the physical life of their sons”. The “physical life of children” started to have a real attention, becoming object of hospital care, as well as of a dedicated medical (pediatric) culture, also due to a growing treatise literature [2]. The first treatise on Pediatrics, *De Infantum Aegritudinibus et Remediis*, was written by the Italian Paolo Bagellardo in 1472. However, childhood medical publishing was lacking in Italy during the seventeenth century. This was related to the political, religious, and cultural events occurred between the end of the 16th and the seventeenth century. The Counter-reform (Trento, 1545–1573) played a substantial breaking action on the scientific progress in the countries of catholic area, with the imposition of the Aristotelian orthodoxy. Consequently, cultural activities in the fields of natural sciences and medicine migrated from southern to northern Europe, especially Netherlands, England, and Scandinavian countries. Indeed, among the most important pediatric treatises of the 17th and 18th centuries stood out those of the English William Harris (*De Morbis Acutis Infantum*, 1689), George Armstrong (1767) and Michael Underwood (1784), and that of the Swedish Nils Rosen von Rosenstein, published in 1765. In 1794 Christoph Girtanner, a Swiss pediatrician, wrote another relevant text, which led the way to the great pediatric treatise literature flourishing during the nineteenth century in France (Billard, 1828; Rilliet and Barthez, 1843; Bouchut, 1845; Comby, 1892), Germany (Vogel, 1860; Gerhardt, 1877; Henoch, 1881; Baginsky, 1882), Russia (Filatov, 1894) and United States of America (Smith, 1869). Italy followed at the end of the nineteenth century with its only pediatric book of that time (Biagini, 1897), also due to the cultural dependence from foreign countries, which finished only after the *Risorgimento* and the process of unification [2].

#### The first schools of pediatrics and children’s hospitals

The first pediatric school was founded in Italy, even if it lasted for a short time. In fact, on April 1802, a chair of pediatrics was born in Florence on initiative of the king of Etruria, Ludwig I of Bourbon, who entrusted the task to Professor Gaetano Palloni, who gave lessons at the *Ospizio degli Innocenti*. The school of Palloni lasted just 3 years, until 1805, when the queen Maria Luisa of Spain suppressed the chair of children’s diseases. However, in 1807, she restored the chair of pediatrics, owing to the high number of deaths among children. Nonetheless, also this time the Florentine School of Pediatrics had no luck. Indeed, due to the upheavals caused by the Napoleonic gestures, all the old institutions were suppressed or renewed and, in the same year (1807) when Tuscany became a French province, the school was definitively closed [1].

While the *Ospizio degli Innocenti* in Florence was the first, although brief, seat of pediatric teaching, the first real pediatric hospital was born in Paris on May 1802, in the middle of the Napoleonic era. The hospital, which was called *Hôpital des enfants malades*, was set up inside an old convent of nuns which was variously used in different times, until, during the Revolution, it became an orphanage (*Maison de l’Enfant Jesus*). It hosted children from 2 to 15 years old. Alongside the hospital wards, an outpatient clinic was also set up for care of patients with less serious diseases and of those who did not need hospitalization. In the same location, activities for teaching and spreading childcare notions to mothers belonging to popular classes were also carried out. The *Hôpital des enfants malades* soon grew as center of pediatric studies and for the spread of pediatric culture during the whole nineteenth century, becoming the cradle of French and European pediatrics [1].

#### Pediatrics and society in France

The French Pediatric School foundation overlaps with that of the *Hôpital des enfats malades*, between the end of 1700 and the beginning of 1800. The prestige of this school is related to great scientists as Bichat, Corvisart, Laennec, who were not pediatricians, but contributed to the advancement of pediatrics [3]. Furthermore, there were other eminent physicians particularly interested in pediatric diseases. Charles Michel Billard (1800–1832), founder of the pediatric pathological anatomy, studied many corpses of children and babies died in the Parisian orphanages. Fréderic Rilliet (1814–1861) and Antoine-Charles-Ernest Barthez (1811–1891), both doctors at the *Sainte Eugénie* Hospital in Paris, published in 1843 the *Traité clinique et pratique des maladies des enfants*, which was the reference text for the pediatricians of the nineteenth century. Eugene Bouchut (1818–1891) was the first to use the laryngeal intubation in the croup (1858). Armand Trousseau (1801–1867) carried out studies on convulsions, chorea, eruptive fevers, diphtheria, and typhus. His fame is related to the first tracheotomies which performed in Paris, defining technique and postoperative treatment. His name is linked to the sign of tetany [4]. Marie-Jules Parrot (1829–1887) was interested in the cerebrovascular lesions of childhood, and studied the nutritional disorders of early infancy, coining the term “atrepsia”. The pseudoparalysis of luetic infants bears his name [5–7]. Pierre Costant Budin (1846–1917) and Adolphe Pinard (1844–1934) were obstetricians and sustained the relevance of boiling milk [8] and breastfeeding, respectively. Thèophile Roussel (1816–1903), was a doctor and politician involved in social and occupational medicine [9]. Jean Bernard Antoine Marfan (1858–1942) was the first professor of Early Childhood Clinic in Paris. He dealt with many fields of children’s pathology and had a great scientific production [10]. In 1881 and 1897 were launched, respectively, the *Monthly Review of Childhood Diseases* and the *Children’s Medicine Archives* [1].

#### Pediatrics and society in Central Europe

German pediatrics started to grow and to be notable during the eighteenth century. In 1753 Jakob Reinbold Spielmann (1722–1783) was the first to analyze the milk of women and domestic animals. In 1787, Joseph J. Mastalier founded in Vienna the first *Public Institute for Sick Children*, which was, rather than a real hospital, an outpatient pediatric clinic. About 50 years later, the first Austrian pediatric hospitals (*Sainte Anne* in 1837, and *Saint Joseph* in 1842) were built in Vienna. Conversely, German pediatrics was officially born in 1830, with the foundation of a small ward at the *Charité Hospital* in Berlin. It developed as a clinic in the following decades, under the direction of Barez. This latter had a prestigious teaching activity and founded the first pediatric journal in the world: the *Journal für Kinderkrankenheit*. Other pediatric hospitals were built throughout the century in the area belonging to ​​Germanic culture, which had in the Viennese Pediatric School its driving force, proof of an increasing interest in childhood. Carl Credé (1819–1892) was obstetrician, and proposed the prophylaxis of blenorrhagic conjunctivitis of the newborn (main cause of neonatal blindness of that time), by the instillation in the conjunctival sac of 2% silver nitrate. Eduard Heinrich Henoch (1820–1910), considered the founder of clinical pediatrics in Germany, was director of the Pediatric Clinic at the *Charité Hospital* in Berlin. His name is linked to the *purpura fulminans*, which from him took the name of “Henoch purpura”. Franz Soxhlet (1848–1926), chemist and physiologist, studied milk sterilization and was able to fractionate its proteins into casein, albumin, globulin and lactoproteins. Alois Epstein (1849–1918) was director of the Prague Brefotrophy, which became with him a great pediatric school. Theodor Escherich (1857–1911) linked his name to the bacteriological research on intestinal germs, and on changes of the intestinal flora in infants with nutritional disorders. He discovered the bacterium *coli*, which was then called *Escherichia coli*. Carl von Pirquet (1874–1929) introduced the concept of allergy. He also supported the suffering childhood, becoming organizer of social provisions for poor children, especially after the terrible famine that struck Austria after the defeat of World War I [1].

#### Pediatrics and society in the United Kingdom

The first interests in childhood diseases, although not yet framed into pediatric schools, began in Great Britain as early as the seventeenth century. Daniel Whistler (1619–1684), and then Francis Glisson (1597–1677), started indeed around 1650 the first studies on rickets, while Thomas Sydenham (1647–1732) deepened various topics of pediatric interest, such as exanthematous diseases, chorea (Sydenham’s chorea), difficult dentition and scurvy [1]. Actually, English pediatrics started with George Armstrong, and was characterized by a greatly humanitarian and sensitive care. In 1769 in London, he made the first generous experiment of pediatric care. Indeed, he opened then a pediatric clinic, where cured about 35,000 children in 12 years, sustaining alone costs and efforts. Andrew Wilson (1718–1792) was his successor, for a short time, in the direction of the London pediatric clinic, which closed in 1783 shortly after the death of its founder, due to lack of benefactors and funds [1, 2].

Before having a real children’s hospital in Great Britain, it was necessary to wait until the second half of the nineteenth century, when the *Great Ormond Street Children’s Hospital* was founded in London in 1852. It may be considered the cradle of English pediatrics, and was the first place where pediatrics was taught. Its first director was Charles West (1816–1898). In 1871, he published *Above some disorders of the nervous system in children*, where he described the infantile myoclonic encephalopathy, which took from him its name (West syndrome). He also gave a significant contribution in the field of the organization of pediatric hospitals, publishing a study entitled *On hospital organization, with special reference to the organization of hospitals for children*. Thomas Barlow (1845–1945) is also linked to the prestigious hospital for sick children in *Great Ormond Street*, where he completed his studies. They were dedicated to infantile scurvy, to which he first gave features of autonomous disease, providing clinical and anatomical-pathological evidence. Scurvy took, then, from him the name of Barlow’s disease. George F. Still (1868–1941) followed in the direction of the *Great Ormond Street Hospital*. He described chronic childhood primary polyarthritis, which was then named Still’s Disease [1, 2].

#### Pediatrics and society in Italy

The first organizations for childhood appeared for the first time in Italy in the medieval age. The first institutions are the *Ospizi* for foundlings or *gettatelli*, which was the name attributed to abandoned or refused newborns. Including the so called “wheels” (which served to receive the abandoned newborns, guaranteeing anonymity to those who left them), they were expression of a charitable attitude, and were able to counteract infanticide [11]. To find a new type of institutes dedicated to childhood, especially sick children, it is necessary to wait the *Ospizi Marini* of the nineteenth century. They were promoted by the intelligent and passionate work of Giuseppe Giannelli, and then of Giuseppe Barellai (1813–1884) [12]. This latter founded the first marine colony in Viareggio in 1862, which was followed by many others in marine and mountainous areas. These institutes arose thanks to the commitment of spontaneous civic committees, and to the awareness of the benefits which children affected with tuberculosis or rickets might have from thalassotherapy. The requirement was that of belonging to poor families, among which, moreover, there were most of the affected subjects [12].In 1843, Count L. Franchi founded the *Regina Margherita Children’s Hospital* in Turin, which was the first Italian pediatric hospital. Soon thereafter, in 1845, the *Ospedaletto di Santa Filomena* was founded by the Marquise Falletti of Barolo in Turin, specifically intended for girls affected with tuberculosis and/or rickets, and aged 3 to 12 years. In 1869, the *Bambino Gesù Hospital* was built in Rome, on the initiative of the Duchess Salviati, where children from 2 to 12 years old were accepted (Table 1) [1, 2].

**Table 1:** The first Pediatric Hospitals in Italy (modified by Burgio GR, 2007 [2])

*City*	*Hospital (Director)*	*Year*
Alessandria	Cesare Arrigo (Prof. Paolo Bosio)	1886
Ancona	Children’s Hospital (Prof. Alberto Caucci)	1900
Bologna	Ospedalino (Dr. Marcellino Venturoli, Dr. Gaetano Modonesi)	1880
Brescia	Umberto I (Prof. Andrea Pagani-Cesa)	1902
Cremona	Children’s Hospital (Dr. Felice Celli)	1881
Cuneo	Regina Elena (Dr. Teresio Cattaneo)	1912
Florence	Anna Meyer (Prof. Giuseppe Mya)	1891
Genoa	S. Filippo (Prof. Luigi Della Valle)	1888
Livorno	Santa Famiglia (Prof. Alberto Funaro)	1888
Lodi	Vittorio Emanuele II (Dr. Oreste Grazia)	1927
Mantova	Bulgarini	1858^a^
Milan	Children’s Hospital (Prof. Girolamo Taccone)	1899
Modena	Pietro Siligardi (Prof. Riccardo Simonini)	1911
Naples	Gesù e Maria (Prof. Francesco Fede)	1881
Palermo	Children’s Hospital (Prof. Rosario Buccheri, Dr. Antonio Carini)	1882
Parma	Children’s Hospital (Prof. Cesare Cattaneo)	1900
Rome	Bambino Gesù (Prof. Francesco Valagussa)	1869
San Remo	A. Nuñez Del Castillo (Dr. Vincenzo Pesante)	1908
Turin	Opera Pia Barolo-Santa Filomena (Dr. Giovanni Battista Filippello)	1845
Turin	Koelliker-Mensi (Prof. Enrico Mensi)	1928
Trento	Maria dei Savoja (Dr. Carlo D’Anna)	1920
Trieste	Spedale Infantile, Burlo Garofolo (Dr. Paolo Israeli)	1867, 1907
Venice	Umberto I (Prof. Ettore Giorgi)	1892
Verona	Alessandri (Prof. Giuseppe Zambelli)	1912

^a^at the beginning occasionally and poorly functioning

However, in many cities, hospital care for children was also provided within large general hospitals, which dedicated special pavilions to them. A critical issue of the hospital care of that time was the exclusion of children under the age of 3, among which there was the greatest morbidity and mortality, due to the difficult management of such young patients. Although a hospital vocation of some orphanages and institutes for children with rickets, near to the twentieth century the united Italy was still widely poor of structures for hospitalization and cure of children, especially infants. Moreover, 80 years passed from the foundation of the Florentine Pediatric School, before seeing the birth, in a united Italy, of a chair of pediatrics. The credit went to Dante Cervesato (1850–1905). After gaining experiences in the pediatric field as student at the *Wiederhofer* of Vienna, he returned to Padua, and was able to set up a small Pediatric Clinic, where he received in 1889 the assignment of full professor of Pediatrics. From Padua, Cervesato moved to Bologna in 1900, where he created a thriving pediatric school. He there performed studies on tetany, infantile tuberculosis, neonatal hemorrhagic diseases, appendicitis, intestinal tumors, liver cirrhosis, and poliomyelitis.

Therefore, also due to a new cultural attitude towards childhood, a change in the field of pediatric care occurred between the end of the 19th and the beginning of the twentieth century. Indeed, also in Italy it became clear that childhood had the right to organized and structured places of cure, based on their specific needs. In 1876, a Children’s Hospital was built in Trieste (named in 1907 *Burlo Garofolo*), then followed by many others, among which Naples and Cremona (1881), Palermo (1882), Genoa and Livorno (1888), Florence (1891), Milan (1897), Bologna (1907) and Modena (1911) (Table 1). Infancy finally received from society a new attention, which never enjoyed before. However, almost all children’s hospitals were funded by private charities, and scarcely by initiative of public institutions. Often these hospitals began their activity in humble rented buildings, to become larger over time with subsequent extensions, restorations of old buildings, or even with construction of new ones [1, 2, 13]. It is noteworthy that some hospital admissions did not have medical indications. They were healthy children “which enter healthy for various reasons … , among which the more frequent is here the concomitant admission of the sick mother in another part of the hospital, or that healed, they stay abandoned for many, painful or shameful, reasons” [14]. This declaration of Ponticaccia (1908) is a complaint for some parental behaviors, as well as a clear proof of the social role which sometimes the hospital had to play. The patients who came to hospitals belonged to the poor classes, which were vulnerable for lacking diets and/or unhealthy environments. The scientific articles which appeared at the end of the nineteenth century and the beginning of the 20th one, provided precious information on the reasons of hospitalizations of those years, and of their length [15–18]. The little patients, especially infants, were often hospitalized for severe conditions of “atrepsia”, characterized by extreme decay of the general conditions, frequently irreversible and fatal [19]. Francesco Fede (1832–1913) first related primitive atrepsia to malnutrition, underlining that these children belonged to the poorest social classes, and calling upon an intervention from authorities aimed at improving their conditions [19]. He was the greatest exponent of early Italian pediatrics, even if chronologically not the first. He was a founding member and president of the Italian Society of Pediatrics, and in 1893 he founded the periodical *La Pediatria* [13]. Subsequently in 1897, Luigi Concetti (1855–1920) introduced Pediatrics as a free course at the University of Rome. He promoted the first Congress of the Italian Society of Pediatrics, of which he was a founding member and then president in 1903. He founded in 1904 the *Journal of Pediatric Clinic*, together with Giuseppe Mya (1857–1911) [20]. Mya was called in 1891 in Florence to hold the chair of Pediatric Clinic. In 1901, he transferred the small rooms of his Institute at the *Maternity Hospital* to the *Anna Meyer Children’s Hospital*, which the Marquis of Montagliani founded in 1887 in memory of his wife (Table 1). In 1916, Vitale Tedeschi (who followed Dante Cervesato in Padua) discussed the possibility to hold the mothers within efficiently and safely organized pediatric wards [21]. It was clear, from the beginning of the foundation of the first pediatric hospitals, that hospitalization might induce suffering in children and parents due to their separation. The time for the introduction of the mother near to her son seems however to be far, but a new attention to the physical and psychological needs of the child was starting to grow.

In more recent years, a diffuse change of point of view towards childhood finally shows the necessity of reconstructing, within hospitals, the binomial mother-child, promoting a specific and global approach to the little patient [22, 23]. During the 1970s a process of de-hospitalization and humanization of pediatric hospitals started, also through the creation of new outpatient systems (Day-Hospital, and then Day-Surgery). In the meantime, and to respond to new and different epidemiological needs, the old sanatoriums for tuberculosis were dismantled or used for other health issues and/or diseases. Then, around the ‘80s, through many regional laws included as aims of health plans, the doors of the hospital wards started to open to the mothers of sick children. Initiatives sustained by associations of families and volunteers were carried out, allowing children to continue the normal activities within hospitals, like games and school, also through psychological support and/or that of cultural mediators. Also, the environments were humanized and tailored on the child. The habit of decorating the walls of infirmaries and hospitalization rooms progressively spread and, in neonatology units, the practice of rooming-in began [24]. In the same years, primary care were carried out by family pediatricians in all the areas of the country.

#### Pediatrics and society in Palermo (Sicily)

The Pediatric Clinic was born in Palermo in 1903, when Rocco Jemma, young and brilliant doctor working in Genoa, was called to hold the role of professor of Pediatrics at the University of Palermo. Promoter of this initiative was Ignazio Florio, belonging to one of the richest and most influential European families of entrepreneurs and patrons of the time. In a few years a new and efficient structure was built, and inaugurated in 1907 [25]. Rocco Jemma founded in those years a real school of pediatricians, who came from all Sicily. When Jemma moved to Naples in 1913, the most brilliant of his pupils, Professor Giovanni Di Cristina, succeeded him. He continued the work of his master, further expanding the scientific activities of the Clinic and starting new social and care initiatives [16]. It is due to him, moreover, the discovery of an effective and decisive treatment for the cure of visceral leishmaniasis with antimony salts, then universally used, which allowed to overcome such infectious disease, commonly lethal until then [17, 26]. He was active for the construction, on lands and with funds obtained from donations, of the hospitals *Casa del Sole* (assigned as tuberculous sanatorium in a hilly area of the town) and *Aiuto Materno* (for the hospitalization of children with high social risk). His premature and unexpected death, in 1928, left a great emptiness in pediatrics of the whole island. The city dedicated to him the Children’s Hospital in 1929, which he wanted closely related to the Pediatric Clinic, and which thereafter took his name. After Di Cristina, the direction of the Clinic and the Hospital passed to La Franca, and then Cannata, Maggiore, and Gerbasi. In 1946, the School of Specialization of Pediatrics was established, favoring the recruitment of scientifically able young doctors. Gerbasi was principal of the Faculty of Medicine and rector of the University, and gave shine to the pediatrics of Palermo, also at a national level. His school was particularly rich of prestigious pupils and personalities, first Giuseppe Roberto Burgio (who later became director of the Pediatric Clinic in Perugia, and then in Pavia). Hematology, infectious diseases, and nutrition were his areas of major scientific impact. He gave decisive contributions on deficiency diseases, as the definition of the perniciosiform anemia of infants (Gerbasi’s anemia), and on dystrophies [26, 27]. The great social caliber of his activities was evident also during the earthquake of Belice in 1968, a dramatic time which Gerbasi faced moving on site doctors, nurses, and hospital equipments.

### Pediatrics and society today

**Fig. 1:**
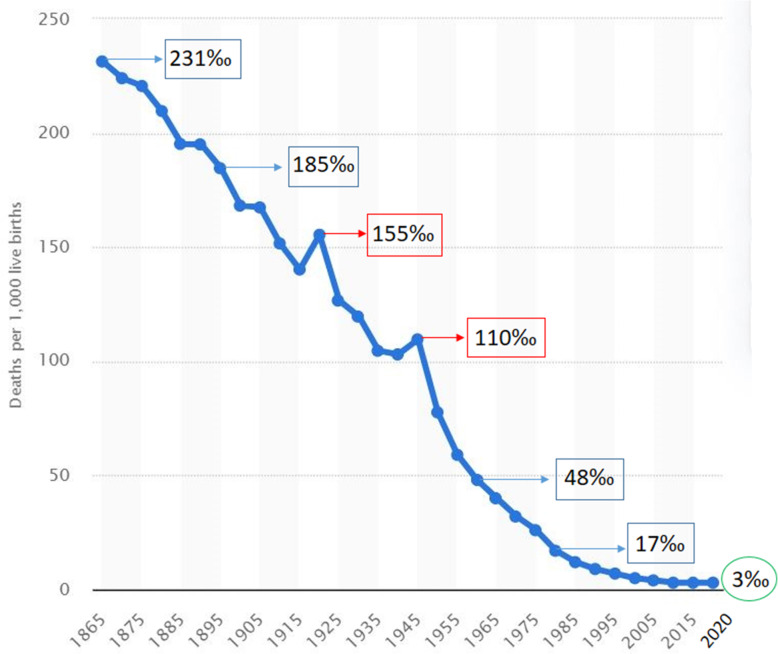
Infant mortality rate (under 1 year old) in Italy, from 1865 to 2020 (modified by O’Neill A, 2019 [28])

The changes that took place over the centuries, and described so far, were innumerable and extraordinary. Society, institutions, and political and economic structures of countries underwent profound transformations. Indeed, the political and health reforms implemented in Italy (and in the other European countries) during the last decades, and the increased economic well-being allowed the reduction of infant mortality rates, which currently are among the lowest worldwide. Specifically, the infant mortality rate for children < 1 year of age (IMR) was 231‰ in 1865, and fell to 185‰ already before the end of the nineteenth century (1895) [28, 29]. Then, this downward trend became even more relevant, till the first two decades of 1900. Afterward, it had two sudden stops and reversals, corresponding to the two war periods. Moreover, the 1920 IMR (155‰) also included the deaths due to the Spanish flu epidemic [29]. Thereafter, in the interwar period (1930), such rate halved (119‰) if compared to the initial recorded values, and dropped below 50‰ in the 1960s and 20‰ in the 1980s, reaching 3‰ between 2015 and 2020 (in Europe decreased from 38.2‰ in 1961 to 3.4‰ in 2019) [28–30] (Fig. 1).

The reduction of IMR over time was associated to a variation of the causes of death. Their analysis better defines the improvements obtained, showing the progressive disappearance of infectious diseases (from about 65% in 1895, to 2% in 2015), and the emergence of other ones, which today mainly includes congenital malformations and conditions of perinatal origin (69%) [29]. Pediatrics significantly contributed to the achievement of these formidable results, through the development of a culture of children’s rights, the acquisition of specific technical knowledge and skills within the context of a constant medical and technological progress, and the control of previously endemic (malaria) and/or past and current communicable diseases (syphilis, tuberculosis and more recently measles, pertussis and lastly COVID-19) [31].

The pediatrician had to adapt and reshape himself in the light of the sociocultural changes of society, as well as of the current biological and psychological features of patients, of what infants, children and adolescents are today [32]. Old diseases disappeared, or their prognosis is significantly improved for ever more effective therapies. New diseases appear, or re-emerge with higher incidence or prevalence, also due to the constant migratory flows from low-income countries to western ones. Diseases which had poor prognosis, are no longer considered as such (oncohematological and genetic ones), in relation to the continuous updating of therapeutic approaches (i.e., transplantations, gene therapies). New techniques of intensive care allow to extremely preterm newborns to survive, and novel treatments (i.e., hypothermia) to reduce adverse outcomes and morbidities. New tools for the identification of genetic diseases (i.e., next generation sequencing) permit more precise diagnosis, prognosis and counselling to patients and their families. New antibiotics, more rationalized treatments, in addition to early and multidisciplinary management, improve quality and length of life of children with complex and chronic diseases [2]. Then, despite the overcoming of many diseases, however pediatricians must increasingly face new health critical issues (new addictions, as the abuse of technological and digital devices [33], contrast to overweight and obesity, care of the migrant child, new infections, as evidenced also by the devastating novel coronavirus pandemic). The functional and environmental changes of pediatric hospitals, as well as the hygienic and structural ones, must be adjusted to the epidemiological mutations of diseases, in addition to the new diagnostic and therapeutic approach to sick children. They must be realized to keep pace with the times, and to guarantee a careful and updated clinical care. Furthermore, to answer to the many and relevant changes of the current digital and hyper-connected society, which especially occurred in recent years, the pediatrician must be formed and equipped with a wide cultural baggage, not exclusively made by eminently clinical and technical aspects. The complexity of his role, in addition to the new scientific knowledge (i.e., the acquisition on the epigenetic mechanisms subtending diseases), led to an increase of his responsibilities, and then to the need to own holistic competences, which should span bioethical, law, relational and communication issues, and several others including pedagogy, bioengineering, sociology, economy, art, sport, politics, technology, music, botany, poetry [34–41]. They compose the cultural background of pediatricians, to take care of all children effectively and competently.

The goal of today’s pediatrician is to protect and improve the health of all children, guaranteeing their fundamental rights from conception. The data relating to social inequalities in our country, as in the whole world, are worrying, especially if we refer to the pandemic period. Although neonatal and child mortality in Italy decreased, notable disparities still remain disadvantaging insular and southern regions (linked to cultural, economic and social factors, in addition to organizational problems also referring to the perinatal network and the high number of small birthing centers) than center-northern ones, and foreign citizens than Italians [31, 42]. Such inequities are amplified if we look at the European context, and even more at the global one. Indeed, there is a significant territorial disparity in the access to health care, as well as in education, and adequate living conditions. Most of these children live in the southern regions of our country (and of the world), where there is a high risk of social exclusion, leading to possible adverse long-term consequences. It is pediatrician’s duty to work to guarantee to every child the same right to health and education, regardless of the family and region of origin [43].

The gradual reduction in funding for the health sector, which characterized the last few decades, led to a profound suffering of our national health system (NHS), which became particularly evident during the pandemic: in a such dramatic time, indeed, local doctors and pediatricians were literally overwhelmed by an immense care burden. Today’s pediatrician must therefore find a new and adequate place within a new structure of the NHS, which must be remodeled and oriented to more effective care networks. Furthermore, the collapse in the number of pediatricians, which will further worsen in the next years, requires the development of a new system able to guarantee pediatric specificity and the right of all subjects in developmental age to be assisted by the pediatrician, with a continuity of care between territory and hospital. Currently, due to lack of specialists in Pediatrics, the child is often evaluated in the first instance by the doctor for adults, with the inevitable risk of clinical inappropriateness. It seems therefore crucial to reformulate university and specialist training programs, supporting the most lacking areas based on territorial needs [43].

Finally, the pediatrician must sustain the cultural and scientific theme of ​​prevention. The promotion of healthy lifestyle (primarily breastfeeding), starting before conception and during the first 1000 days, represents the most effective intervention to counteract the development of chronic socially communicable diseases (i.e., obesity, diabetes, cardiovascular diseases), which today represent among the main causes of morbidity and mortality also among children. For this purpose, all health education activities may play a key role. Investing in the school, indeed, as well as in the health system and in policies to support families, will likely reduce inequality, educational poverty, social neglect, behavioral disorders, delinquency, and ultimately many of the health problems of the children of today and tomorrow [44].

## Conclusions

Pediatrics arose from the need, developed in different parts of the world and in different times, and not necessarily felt by doctors, to protect children. Many pediatric hospitals were born thanks to sensitivity and attention of people animated by authentic philanthropy and altruism. The peculiarity of pediatrics, as a global science immersed in social aspects, outlined its uniqueness and specificity since its birth [45]. Although more than 200 years passed since its foundation, and despite the extraordinary changes in the society, the advances and acquisitions obtained in the medical and technological fields (e.g., treatment and control of many diseases, reduction of IMR), however spirit, values, and targets which the pediatrician sets in the everyday work, are still unchanged. Aims of today’s pediatrician are, in fact, care of children with the best possible assistance, protection of their rights within families and in the society, fight against inequalities and poverty, and respect for their identity in all its forms, personal, cultural, and religious.

The pediatrician must take care, indeed, of children’s health, considered as mental, physical, and social well-being. He has then to cure, as well as to promote such well-being. Indeed, his responsibility goes beyond the simplicity of the doctor-patient relationship. It involves several people, all those who take care of newborns, children, adolescents. This involvement may also refer to teachers, coaches, friends, with a *domino* effect which reaches the entire world around the single child [45]. The modulation of all these relationships makes clear the social role of pediatricians. Moreover, we know today those lifestyles and quality of life can build the well-being through personal choices, and above all that such well-being is the result also of the environment where we live. Therefore, the pediatrician has a responsibility on families’ decisions, and in the meantime, he must be aware of politically and socially acting to favor the presence of the best possible context oriented to promote the well-being of the child.

## Data Availability

Data and materials used and analyzed during the current study are available from the corresponding author on reasonable request.

## Abbreviations

COVID-19: Coronavirus disease 19
IMR: Infant mortality rate
NHS: National health system
